# Development and validation of a predictive risk model for post-stroke deep vein thrombosis

**DOI:** 10.17305/bb.2026.14312

**Published:** 2026-06-14

**Authors:** Baichen Wang

**Affiliations:** 1 Department of Neurology, Tongde Hospital of Zhejiang Province, Hangzhou, Zhejiang, China

**Keywords:** Acute ischemic stroke, deep vein thrombosis, nomogram, risk stratification

## Abstract

Deep vein thrombosis (DVT) represents a common and potentially fatal complication following acute ischemic stroke (AIS). However, early risk stratification is often difficult due to the asymptomatic nature of post-stroke DVT and the scarcity of AIS-specific predictive instruments. This study aimed to develop a nomogram utilizing baseline patient characteristics to forecast DVT risk after AIS. In this retrospective study, 848 AIS patients were analyzed, with 79 subsequently developing DVT. Least Absolute Shrinkage and Selection Operator (LASSO) regression and multivariable logistic regression identified five independent predictors: age (odds ratio [OR] ═ 1.074), fibrinogen (OR = 1.700), D-dimer (OR = 1.123), platelet count (OR = 1.001), and sex (OR = 0.475). A predictive nomogram was then constructed based on these variables. The model achieved an initial area under the receiver operating characteristic curve (AUC) of 0.809 and a Brier score of 0.074. Internal validation, conducted via 1,000-resample bootstrapping, yielded a stable optimism-corrected AUC of 0.800. Calibration plots demonstrated excellent concordance between predicted probabilities and observed outcomes. Decision curve analysis (DCA) indicated a positive net clinical benefit across a threshold probability range of 1%–91%, encompassing the clinically relevant 5%–20% decision window. In conclusion, this study successfully developed an exploratory nomogram for the early estimation of DVT risk in patients with AIS.

## Introduction

Acute ischemic stroke (AIS) is a leading cause of global mortality and disability [1–3]. Deep vein thrombosis (DVT) represents a common and severe complication following AIS [4, 5]. Dislodged DVT can lead to fatal pulmonary embolism, accounting for approximately 25% of early stroke-related mortality. Clinical evidence indicates that the incidence of DVT in patients with AIS ranges from 18.0% to 23.5% [6–8], a rate that can reach 75% in patients with severe hemiplegia. Consequently, early identification and prevention of DVT are crucial for reducing mortality and improving clinical outcomes in affected patients.

Currently, color Doppler ultrasound is the first-line noninvasive diagnostic modality for lower extremity DVT [9, 10]. However, post-stroke DVT is often asymptomatic [11]. Furthermore, patients with AIS frequently experience cognitive impairment or aphasia, neurological deficits that hinder their ability to effectively report early symptoms such as leg pain or swelling. This often results in missed diagnoses and delayed interventions [12, 13]. Routine venous thromboembolism risk assessment tools, such as the Caprini score, lack AIS-specific variables, leading to insufficient specificity when applied to the stroke population [8]. Given that DVT development is a multifactorial process, comprehensive risk prediction models specifically tailored to patients with AIS remain limited.

Nomograms are visual predictive tools that integrate multiple risk factors, proving valuable for prognostic evaluation and risk stratification across various diseases [14]. In this study, we systematically collected demographic, clinical, laboratory, and treatment data from patients with AIS to identify independent risk factors for lower extremity DVT. Utilizing these factors, we developed and validated a nomogram for DVT prediction. This tool aims to facilitate the early identification of high-risk patients, guide individualized thromboprophylaxis strategies, and ultimately improve clinical outcomes.

## Materials and methods

### Study design and population

This was a single-center retrospective observational study analyzing the clinical data of 848 patients hospitalized at Tongde Hospital of Zhejiang Province between January 2021 and December 2025 who underwent venous ultrasound evaluation. Inclusion criteria were: (1) diagnosis of AIS based on current clinical guidelines [13]; and (2) completion of bilateral lower-extremity venous Doppler ultrasound within 14 days of symptom onset. Exclusion criteria included: (1) failure to undergo bilateral lower-extremity venous ultrasound; (2) presence of DVT at admission or a history of venous thromboembolism; (3) history of lower-extremity fracture, trauma, or conditions causing limb dysfunction; (4) severe hepatic or renal dysfunction, or known coagulopathy; (5) end-stage disease or severe systemic comorbidities; and (6) incomplete clinical data or missing key laboratory parameters.

### Diagnosis of DVT

DVT was diagnosed using bilateral lower-extremity venous color Doppler ultrasound. Diagnostic criteria for DVT included the presence of solid intraluminal echoes, non-compressible venous segments, filling defects, or a complete absence of blood flow signals. Based on these imaging findings, patients were categorized into a DVT group and a non-DVT group. Patients diagnosed with either proximal or distal DVT were grouped and analyzed together as a single composite outcome, regardless of anatomical location.

### Data collection

Demographic and clinical data extracted included age, sex, and comorbidities (coronary heart disease, atrial fibrillation, diabetes mellitus, hypertension, and previous malignancy). Results from initial peripheral blood and coagulation tests performed upon admission were also collected. Laboratory parameters included fibrinogen, D-dimer, activated partial thromboplastin time (APTT), international normalized ratio (INR), thrombin time (TT), prothrombin time (PT), white blood cell (WBC) count, neutrophil count and percentage (Neu, NEUT%), lymphocyte count and percentage (Lym, LYMPH%), monocyte count and percentage (Mono, MONO%), and platelet count (PLT). All tests were performed in the hospital’s central clinical laboratory using standardized protocols. To evaluate systemic immune-inflammatory status, five composite indices were calculated from absolute peripheral blood cell counts: (1) neutrophil-to-lymphocyte ratio (NLR = Neu / Lym); (2) platelet-to-lymphocyte ratio (PLR = PLT / Lym); (3) monocyte-to-lymphocyte ratio (MLR = Mono / Lym); (4) systemic immune-inflammatory response index [SIRI ═ (Neu × Mono) / Lym]; and (5) systemic immune-inflammation index [SII ═ (PLT × Neu) / Lym].

### Ethical statement

This retrospective study was conducted in accordance with the Declaration of Helsinki and approved by the Ethics Committee of Tongde Hospital of Zhejiang Province (Approval No.: 2026-031-JY). Given the anonymous nature of the retrospective data collection, the requirement for informed consent was waived by the ethics committee.

### Statistical analysis

All statistical analyses were performed using R software (version 4.2.1, R Core Team, Vienna, Austria) and Statistical Package for the Social Sciences (SPSS) 25.0 (IBM Corp., Armonk, NY, USA). A two-sided *P* < 0.05 was considered statistically significant. Missing values in the original dataset were handled using multiple imputation by chained equations (MICE) prior to variable selection and modeling. Categorical variables were presented as frequencies and percentages [n (%)] and compared using Pearson’s χ^2^ test. Normally distributed continuous variables were expressed as mean ± standard deviation (x- ± s) and analyzed using independent-sample *t*-tests. Non-normally distributed variables were reported as medians with interquartile ranges [Median (IQR)] and compared using the Mann-Whitney *U* test.

Given 79 DVT events and 26 initial candidate predictors, the Events Per Variable (EPV) ratio was approximately 3.04. Due to this relatively low EPV, the Least Absolute Shrinkage and Selection Operator (LASSO) algorithm was employed for variable dimension reduction. Features with non-zero coefficients were included in a multivariable logistic regression model to identify independent predictors and calculate odds ratios (OR). Variables with a variance inflation factor (VIF) < 5 were retained to avoid multicollinearity. A nomogram was then constructed to estimate the individualized probability of DVT in patients with AIS. The area under the receiver operating characteristic curve (AUC) was calculated to evaluate model discrimination. To assess model reliability and mitigate overfitting, internal validation was performed using 1,000-resample bootstrapping on the final fixed logistic regression model to derive the optimism-corrected AUC. Model calibration was assessed using the Hosmer-Lemeshow test, Brier score, bootstrap-corrected calibration curves, and mean absolute error (MAE). Finally, decision curve analysis (DCA) was performed to evaluate the clinical net benefit of the nomogram across a range of threshold probabilities.

## Results

### Variable selection

The study flowchart is presented in Figure 1. A total of 848 patients were included in this study, of whom 79 (9.3%) developed DVT. As shown in Table 1, baseline characteristics revealed significant differences between the DVT and non-DVT groups in age, sex, and multiple laboratory indices (all *P* < 0.05). LASSO was applied for feature selection to mitigate overfitting (Figure 2A). Using 10-fold cross-validation, the penalty parameter at one standard error of the minimum binomial deviance was selected (Figure 2B). This process identified five predictors with non-zero coefficients: age, fibrinogen, D-dimer, platelet count, and sex.

**Table 1:** Baseline characteristics and values of the study variables

**Variables**	**Total Cohort**	**Non-DVT Group**	**DVT Group**	**Z/t/χ2**	***P* value**
	**(*n* ═ 848)**	**(*n* ═ 769)**	**(*n* ═ 79)**		
*Demographics*					
Age (years)	71 [62,80]	69 [60,79]	79 [73,86]	−6.563	<0.001*
Sex (Male, *n*, %)	526 (62%)	489 (63.6%)	37 (46.8%)	8.537^a^	0.003*
*Clinical features (n, %)*					
Coronary heart disease	146 (17.2%)	129 (16.8%)	17 (21.5%)	1.131^a^	0.288
Atrial fibrillation	99 (11.7%)	77 (10%)	22 (27.8%)	22.1^a^	<0.001*
Diabetes mellitus	312 (36.8%)	282 (36.7%)	30 (38%)	0.052^a^	0.819
Hypertension	655 (77.2%)	597 (77.6%)	58 (73.4%)	0.724^a^	0.395
Malignancy	46 (5.4%)	39 (5.1%)	7 (8.9%)	2.005^a^	0.157
*Laboratory findings*					
Fibrinogen (g/L)	2.69 [2.25,3.53]	2.64 [2.22,3.39]	3.48 [2.64,4.21]	−5.377	< 0.001*
D-dimer (mg/L)	0.47 [0.24,1.17]	0.44 [0.23,0.97]	1.32 [0.57,3]	−6.592	< 0.001*
APTT (s)	27.4 ± 3.1	27.3 ± 3.1	27.4 ± 3.2	0.254	0.799
INR	0.99 [0.95,1.08]	0.99 [0.95,1.07]	1.02 [0.97,1.13]	−3.042	0.002*
TT (s)	16.6 [15.8,17.5]	16.6 [15.8,17.5]	16.6 [16,17.5]	−0.564	0.573
PT (s)	11.4 [10.9,12]	11.3 [10.8,11.9]	11.5 [11.1,12.3]	−2.678	0.007*
WBC (× 10^9^/L)	7 [5.3,9.8]	7 [5.2,9.8]	7.4 [5.7,10]	−0.734	0.463
Neutrophil count (× 10^9^/L)	4.8 [3.3,7.2]	4.8 [3.2,7.1]	5.5 [3.7,8.3]	−1.581	0.114
NEUT% (%)	67.5 [57.5,76.9]	67.1 [57.2,76.5]	72.3 [62.8,81.5]	−2.437	0.015*
Lymphocyte count (× 10^9^/L)	1.5 [1.1,2.3]	1.5 [1.1,2.3]	1.5 [1, 2]	−1.323	0.186
LYMPH% (%)	22.8 [14.6,32]	23 [14.8,32.3]	18.8 [11.9,27.8]	−1.978	0.048*
Monocyte count (× 10^9^/L)	0.5 [0.4,0.7]	0.5 [0.4,0.7]	0.5 [0.3,0.6]	−1.288	0.198
MONO% (%)	6.8 [5.4,8.6]	6.9 [5.5,8.7]	6.4 [4.6,7.7]	−2.243	0.025*
PLT (× 10^9^/L)	214 [156,276]	213 [155,274]	233 [162,291]	−1.749	0.08
*Inflammatory indices*					
NLR	3.18 [1.92,5.39]	3.08 [1.9,5.3]	4.07 [2.44,6.06]	−2.465	0.014*
PLR	137.14 [86.81,210]	135.71 [85.52,203.64]	157.42 [110.59,264.29]	−2.694	0.007*
MLR	0.31 [0.21,0.5]	0.31 [0.21,0.5]	0.35 [0.22,0.5]	−0.625	0.532
SIRI	1.53 [0.77,3.32]	1.48 [0.77,3.27]	1.84 [0.8,3.64]	−1.416	0.157
SII	650.14 [335.72,1227.4]	633.03 [344.93,1171.51]	805.6 [512.06,1661.83]	−3.079	0.002*

Continuous variables are expressed as mean ± standard deviation (x±s) or median [interquartile range, IQR]. Categorical variables are presented as frequency and percentage (n, %). Superscript ‘a’ indicates values obtained using the χ^2^ test. Statistical significance is indicated by * (*P* < 0.05). **Abbreviations:** APTT, activated partial thromboplastin time; DVT, deep vein thrombosis; INR, international normalized ratio; LYMPH%, lymphocyte percentage; MLR, monocyte-to-lymphocyte ratio; MONO%, monocyte percentage; n, number of patients; NEUT%, neutrophil percentage; NLR, neutrophil-to-lymphocyte ratio; PLR, platelet-to-lymphocyte ratio; PLT, platelet count; PT, prothrombin time; SII, systemic immune-inflammation index; SIRI, systemic immune-inflammatory response index; TT, thrombin time; WBC, white blood cell count; χ^2^, chi-square test statistic.

**Figure 1.**
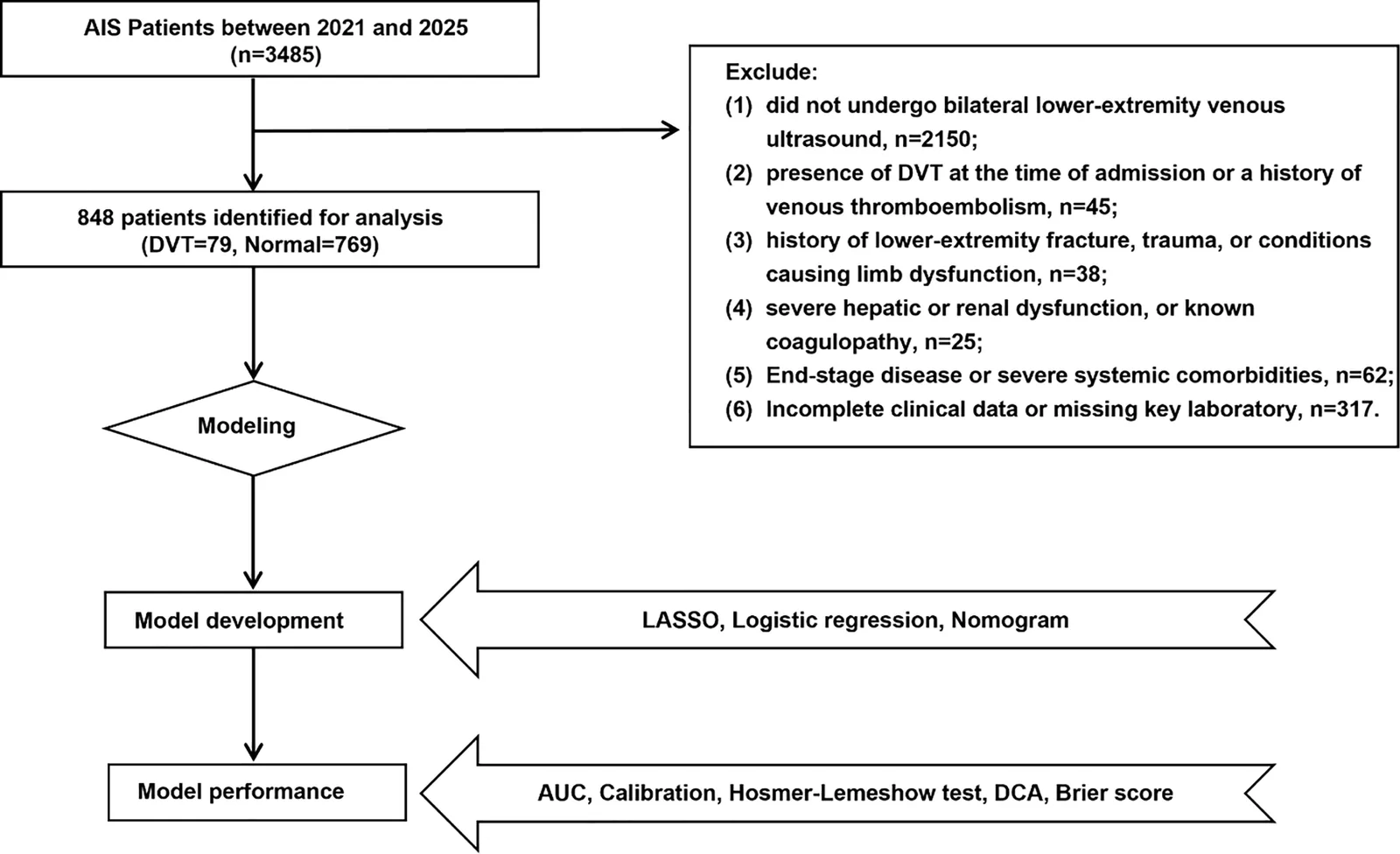
**Flow diagram of study design.Abbreviations:** AIS, acute ischemic stroke; AUC, area under the receiver operating characteristic curve; DCA, decision curve analysis; DVT, deep vein thrombosis; LASSO, least absolute shrinkage and selection operator; n, number of patients.

**Figure 2.**
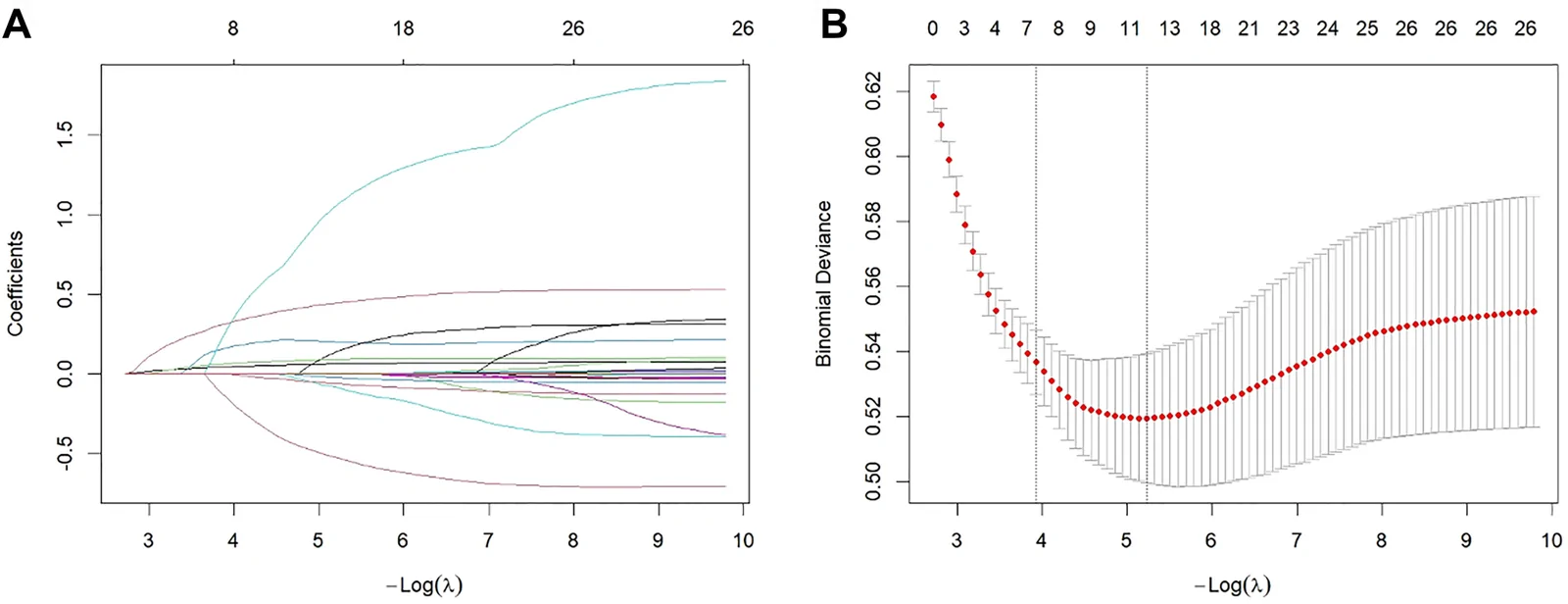
**Variable selection using the LASSO logistic regression model.** (A) Coefficient profiles of candidate variables across different values of the regularization parameter λ. (B) Ten-fold cross-validation for selection of the optimal λ in the LASSO model. The dotted vertical lines indicate the λ value with the minimum cross-validated binomial deviance and the largest λ within one standard error of the minimum. **Abbreviation:** LASSO, least absolute shrinkage and selection operator.

### Nomogram development

The five variables selected by LASSO were incorporated into a multivariable logistic regression model. VIF values ranged from 1.002 to 1.056, indicating an absence of significant multicollinearity. The analysis confirmed all five variables as independent predictors for DVT in patients with AIS (all *P* < 0.05): age (OR = 1.074), fibrinogen (OR = 1.700), D-dimer (OR = 1.123), platelet count (OR = 1.001), and sex (OR = 0.475) (Table 2). Based on these independent predictors, a nomogram was constructed to estimate individualized DVT risk (Figure 3). The multivariable logistic regression equation is formulated as follows: Logit(*P*) ═ –9.540 + 0.072 × Age + 0.531 × Fibrinogen + 0.116 × D-dimer + 0.001 × Platelet Count − 0.745 × Sex. The individualized probability of the outcome can be calculated using the formula: *P* ═ 1 / (1 + exp(−Logit(*P*))). In this equation, categorical variables were coded as follows: Sex was coded as 0 for female (corresponding to “No” in the nomogram) and 1 for male (corresponding to “Yes” in the nomogram).

**Table 2:** Multivariable logistic regression analysis and multicollinearity diagnostics

**Variables**	**β Coefficient**	**Standard error**	**Wald χ2**	**OR**	**95% CI for OR**	***P* value**	**VIF**
Age	0.072	0.013	32.183	1.074	1.048–1.101	<0.001*	1.056
Fibrinogen	0.531	0.104	26.150	1.700	1.387–2.084	<0.001*	1.045
D-dimer	0.116	0.038	9.491	1.123	1.043–1.208	0.002*	1.054
Platelet count	0.001	0.001	6.364	1.001	1.000–1.003	0.012*	1.002
Sex	−0.745	0.263	8.015	0.475	0.284–0.795	0.005*	1.030

*Statistical significance at *P* < 0.05. **Abbreviations:** CI, confidence interval; OR, odds ratio; VIF, variance inflation factor. β, regression coefficient; χ^2^, chi-square statistic.

**Figure 3.**
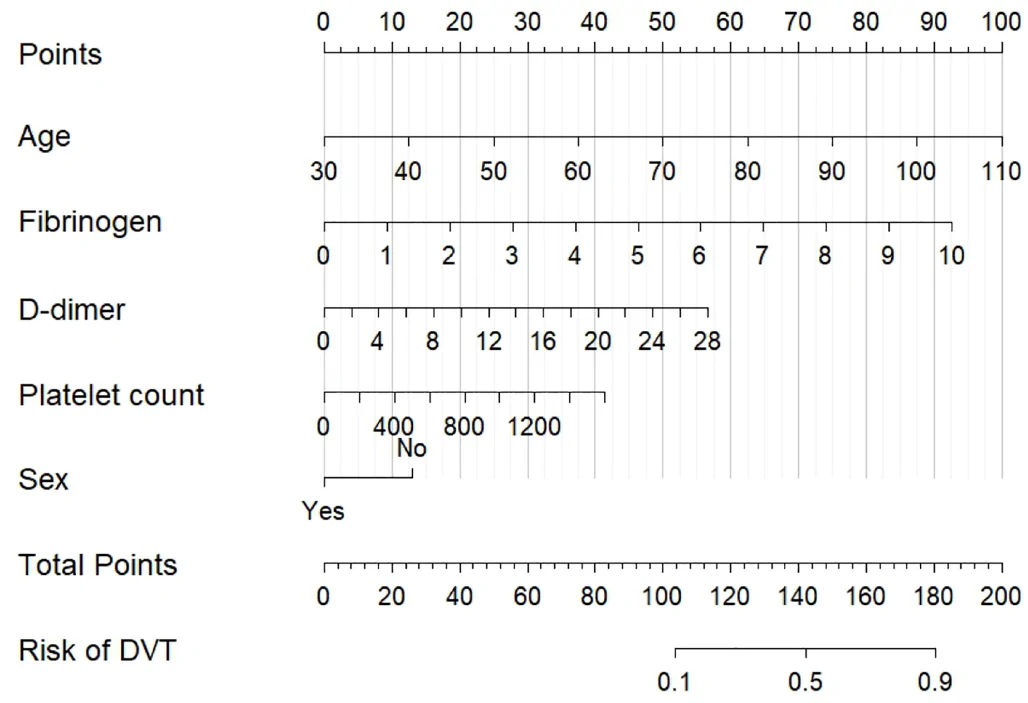
**Nomogram for estimating the individualized risk of DVT in patients with AIS.** Points are assigned for each independent predictor, including age, fibrinogen, D-dimer, platelet count, and sex. The points for all predictors are summed to obtain the total points, which correspond to the predicted probability of DVT. Sex was coded as 0 for female (“No”) and 1 for male (“Yes”). **Abbreviations:** AIS, acute ischemic stroke; DVT, deep vein thrombosis.

### Model performance

The predictive performance of the nomogram was first evaluated in the study cohort. The model demonstrated excellent discrimination, with an AUC of 0.809 (95% confidence interval [CI]: 0.763–0.855; Figure 4A). The Hosmer-Lemeshow test indicated a good fit (*P* ═ 0.896), and the overall model accuracy was confirmed by a low Brier score of 0.074. Following 1,000 resamples bootstrap correction, the calibration curve aligned closely with the ideal diagonal line. The MAE was 0.011 (Figure 4B). Furthermore, the optimism-corrected calibration intercept was −0.080 and the calibration slope was 0.952, indicating good calibration of the model. DCA showed that using the nomogram provided a greater net clinical benefit than either the “treat-all” or “treat-none” strategies across a threshold probability range of 1% to 91% (Figure 4C).

**Figure 4.**
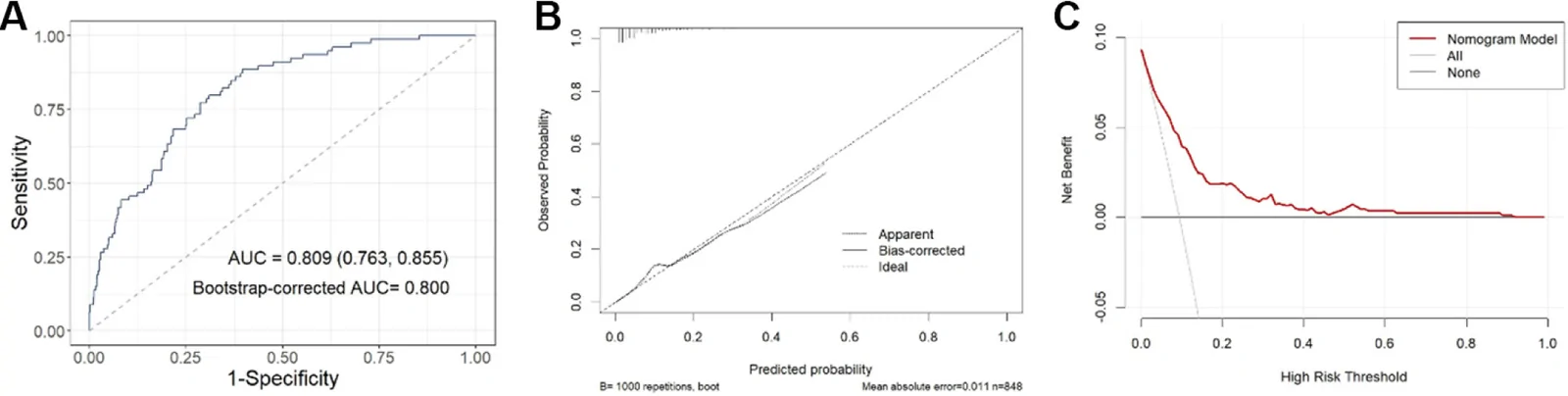
**Performance evaluation of the nomogram for predicting DVT in patients with AIS.** (A) ROC curve showing model discrimination, with an AUC of 0.809 (95% CI, 0.763–0.855) and a bootstrap-corrected AUC of 0.800. (B) Calibration curve after 1,000 bootstrap resamples, showing the agreement between predicted and observed probabilities. The apparent and bias-corrected curves are shown in relation to the ideal reference line. (C) Decision curve analysis (DCA) showing the net clinical benefit of the nomogram compared with the “treat-all” and “treat-none” strategies across threshold probabilities. **Abbreviations:** AIS, acute ischemic stroke; AUC, area under the receiver operating characteristic curve; CI, confidence interval; DCA, decision curve analysis; DVT, deep vein thrombosis; ROC, receiver operating characteristic.

## Discussion

In this study, a nomogram was developed to predict DVT in patients with AIS. Using LASSO and multivariable logistic regression, five independent predictors were identified: age, fibrinogen, D-dimer, platelet count, and sex. The nomogram demonstrated favorable discriminative performance, achieving an AUC of 0.809. The calibration curve closely aligned with the ideal diagonal line, indicating a low degree of overfitting. Furthermore, DCA confirmed a positive net clinical benefit. These findings support the nomogram as a preliminary exploratory tool for the early estimation of DVT risk in AIS patients.

The results indicate that age (OR = 1.074) is the strongest independent predictor for DVT, aligning with previous literature. A multicenter study by Cai et al. [15] reported a substantial increase in DVT risk with every 10-year increment in age among stroke patients. Similarly, Wang et al. [16] found that advancing age leads to reduced venous elasticity and physiologically elevated coagulation factors, promoting a hypercoagulable state. This study further confirms age as a continuous risk factor, emphasizing the need for dynamically heightened clinical surveillance as patients age.

D-dimer (OR=1.123) and fibrinogen (OR=1.700), representing core markers of the coagulation-fibrinolytic system, were also key predictors. Previous studies [17, 18] confirmed that elevated D-dimer levels are an independent risk factor for post-stroke DVT. The degree of D-dimer elevation is associated with the severity of trauma, inflammatory response, and other factors [19]. In surgical or orthopedic trauma, D-dimer elevation primarily stems from direct endothelial damage and postoperative bleeding. However, in AIS, ischemic brain tissue releases abundant tissue factor into the circulation, subsequently triggering a systemic coagulation cascade [20]. Similarly, elevated fibrinogen directly promotes plasma hypercoagulability, confirming their combined critical roles in the thrombotic mechanisms of stroke patients. Furthermore, beyond the traditional view attributing post-stroke DVT merely to limb immobility and local inflammation, recent studies [21] highlight an intrinsic shared pathophysiology between arterial and venous thrombosis. Generalized endothelial dysfunction, systemic hypercoagulability, and overlapping inflammatory pathways serve as a critical bridge linking these events.

Additionally, sex and platelet count were identified as independent predictors. In the cohort, males had an OR of 0.475, implying a relatively higher DVT risk in female AIS patients. This observation is consistent with literature indicating that hormonal variations and differences in lower extremity venous hemodynamics may render females more susceptible to venous thromboembolism [22, 23]. Furthermore, although the continuous variable of platelet count exhibited an OR near 1.001, its inclusion remains biologically plausible. Platelet activation and aggregation are fundamental steps in venous thrombosis, particularly when synergized with stroke-induced release of inflammatory mediators [24, 25].

A recent nomogram developed by Chen et al. [26] demonstrated good predictive value but notably lacked the inclusion of D-dimer due to regional testing limitations, thereby missing a crucial thrombotic biomarker. Conversely, Fang et al. [8] combined the Caprini Risk Assessment Model with D-dimer and fibrinogen, achieving a highly accurate AUC of 0.947. However, the Caprini Risk Assessment Model requires manual, detailed evaluation of numerous clinical variables, which can be subjective and time-consuming in an acute stroke setting. Similarly, recently externally validated models incorporate clinical physical assessments like lower limb muscle strength or Padua scores, which depend on physician evaluation and may introduce subjective bias [27]. In contrast, a major practical advantage of the present model lies in its real-world clinical applicability. The nomogram is exclusively derived from objective baseline clinical characteristics (age, sex) and routine laboratory hematological indices (D-dimer, fibrinogen, platelet count) obtained immediately upon admission. By eliminating the need for complex imaging parameters, specialized biomarkers, or subjective clinical scoring scales, the model significantly reduces clinical workload. This objective, cost-effective, and readily accessible profile allows the model to serve as a rapid screening tool, making it particularly suitable for early risk stratification, even in resource-limited clinical settings. Compared with traditional risk scales, the nomogram directly utilizes original continuous variables, thereby maximizing the retention of individualized prognostic information. The DissolVE-2 study [28] reported a high venous thromboembolism risk in 53.4% of surgical patients and 36.6% of medical patients. In the current model, predicted probabilities clustered between 0.1 and 0.5, aligning well with the actual clinical incidence of DVT in AIS. Consequently, the upper limit of the model’s predicted probability was approximately 0.6, which effectively mitigated the risk of overfitting to unrealistic prevalence rates. Clinically, prophylactic anticoagulation during the acute phase of stroke carries a severe risk of secondary intracranial hemorrhage. Therefore, clinicians must carefully balance thrombotic and hemorrhagic risks. The threshold for initiating routine DVT prophylaxis typically ranges from 5% to 20%. The DCA confirmed a positive net benefit across an exceptionally broad threshold range of 1% to 91%, demonstrating the nomogram’s robust validity across diverse clinical scenarios. Physicians can dynamically calculate an individual’s DVT risk probability upon admission to weigh against their competing risk of hemorrhagic transformation. Particularly within the pragmatic 5% to 20% clinical decision window, initiating thromboprophylaxis for patients exceeding the nomogram-predicted threshold significantly outperforms blind “treat-all” or “treat-none” strategies.

### Limitations

This study has several limitations. First, the model was derived from a single-center retrospective cohort with a relatively small number of DVT events (*n* ═ 79). Due to the retrospective nature of this study, universal DVT ultrasound screening was not feasible. Screening was largely driven by clinical suspicion rather than universal screening, which inevitably introduces potential detection bias. Second, due to sample size constraints, this study lacks an independent validation cohort. Although 10-fold cross-validation and 1,000-resample bootstrap correction demonstrated internal stability, external validation across large-scale multicenter cohorts is essential to verify the model’s generalizability before it can be safely implemented in clinical routines. Finally, owing to missing or inconsistent records in the retrospective database, this study failed to incorporate several major stroke-related predictors, such as stroke severity (e.g., NIHSS score), definite immobility status, and the precise use of prophylactic treatments. Consequently, the potential for residual confounding remains. Future prospective studies must integrate these crucial determinants to better define the model’s applicability.

## Conclusion

Age, fibrinogen, D-dimer, platelet count, and sex were identified as independent predictors for DVT following AIS. Based on these variables, a preliminary predictive nomogram was developed. Although this tool demonstrates potential in identifying high-risk patients to support early clinical decision-making, it fundamentally remains an exploratory model. Future prospective studies across independent multicenter cohorts are required to confirm its generalizability.

## Data Availability

The data presented in this study are available on request from the corresponding author.
